# Nursing practice in relation to older people’s fundamentals of care in nursing homes: An exploratory design

**DOI:** 10.1016/j.ijnsa.2025.100346

**Published:** 2025-05-06

**Authors:** O.M. Nordaunet, E.R. Gjevjon, H. Aagaard, C. Olsson, G. Borglin

**Affiliations:** a Karlstad University, Institute of Health Sciences, Department of Nursing. Universitetsgatan 2, 651 88 Karlstad, Sweden; b Lovisenberg Diaconal University College, Department of Bachelor Education (Nursing). Lovisenberggata 15B, NO-0456 Oslo, Norway; c UiT The Arctic University of Norway, Campus Harstad, Faculty of Health Sciences, Havnegata 5, 9404 Harstad, Norway

**Keywords:** Descriptive statistic, Direct observations, Multimethod, Nurse, Nursing homes, Nursing process, Registered nurses

## Abstract

**Background:**

Nursing practice addressing the physical, psychosocial, and relational needs of older people – the three core dimensions of the fundamentals of care framework along with its overarching dimension, commitment to care – is a complex yet vital aspect of nurses’ scope of practice. However, it is underrepresented in the clinical context of facility-based care, such as nursing homes. Consequently, there is limited understanding of to what extent nurses engage in activities targeting older people’s fundamentals of care needs, the applicability of the framework in practice, and what acts as contextual modulators. Furthermore, contextual modulators of practice require greater attention, especially within increasingly complex healthcare systems, where nursing practice should be studied as a part of a larger system.

**Objective:**

To explore nursing practice, its contextual modulators, and the clinical decision-making processes, as aligned with the nursing process of nurses targeting older people’s fundamentals of care needs in nursing homes.

**Design:**

An exploratory study.

**Setting:**

Four nursing homes across three Norwegian municipalities.

**Methods:**

Structured direct observations were conducted. Thus, observations was supported by a protocol developed from established theoretical frameworks and concepts identified in the nursing literature as relevant to practice or as modulators of practice. Data analysis incorporated both textual and numerical analyses in a multimethod approach.

**Results:**

A total of 4351 framework activities were observed during 411 sessions (189 hours). On average, nurses engaged in 10.58 activities per observation, often addressing multiple dimensions of the framework simultaneously. Activities related to the dimension *commitment to care* were less frequently observed than those in the other three dimensions. We found that most observations showed nurses initiating care with activities targeting *physical needs*, which often expanded to include psychosocial and relational dimensions*.* Registered nurses primarily focused on the assessment phase of the nursing process. Nursing practice was found to be influenced by a lack of risk management, an unsupportive working environment, and unclear leadership and management of care.

**Conclusion and implications:**

This is one of the first studies exploring nursing practice targeting the fundamentals of care framework in this context. We have highlighted the intricate nature of nursing practice, its relationship with clinical decision-making processes, and the functional and performance levels of nursing activities. Contextual modulators were found to negatively influence nursing practice, suggesting the need for improved risk management, a supportive work environment, and clear nursing leadership.

**What is already known**

- Researchers on nursing clearly acknowledge that certain areas of practice, such as the fundamentals of care, are prone to neglect, left undone, or not prioritised by nurses.

- A vital component of nursing practice is nurses’ clinical decision-making abilities, frequently described as the foundation of all nursing care and an integral aspect of autonomous nursing practice.

- Recent recommendations for health service research emphasise the importance of exploring and understanding services – such as nursing practice – as system events.

- Nursing practice is thus deemed susceptible to contextual modulators at both individual and systemic levels, which influence and adjust clinical practice and, consequently, the quality of care.

Alt-text: Unlabelled box

**What this paper adds**

- Cues across all four core dimensions of the framework were observable in nursing practice, although activities within the *commitment to care* dimension were less frequently observed than activities within the other dimensions.

- Observations indicated that the nurses often engaged in several fundamentals of care activities simultaneously within its core dimensions, highlighting the intricate nature of nursing practice in the nursing home context.

- In most cases, nurses initially focused on activities within the framework’s domain of *physical needs*, which quickly expanded to include activities within the *psychosocial needs* and *relational needs* dimensions.

- Registered nurses’ clinical decision-making activities, aligned with the five steps of the nursing process, were primarily focused on the assessment phase.

- Observations of individual and organisational contextual modulators highlighted a nursing practice shaped by a lack of risk management, the absence of supportive working environments, and unclear nursing leadership, as well as management of care.

Alt-text: Unlabelled box

## 1 Introduction

Nursing practice centred on the fundamentals of care is an integral component of nursing (Zwakhalen et al., 2018). However, the empirical foundation guiding nurses has been criticised as inadequate, often leaving them reliant on tradition and guesswork (Richards et al., 2018). The renewed focus on the fundamentals of care, alongside the introduction of a theoretical framework, aims to reaffirm the essence of nursing practice. This approach emphasises the value of nursing through a commitment to care, characterised by a trusting nurse–patient relationship, and the fulfilment of patients’ physical, psychosocial, and relational needs (Feo et al., 2018; cf. Kitson et al., 2013).

The framework (hereinafter framework and fundamentals of care will be used interchangeable) is crucial, as unfinished nursing care (Chiappinotto et al., 2022), particularly in relation to fundamentals of care needs, has been linked to patient safety failures and adverse events mainly in the hospital context (Ball et al., 2014, 2018). This is particularly evident in cases where fundamental care needs have not been consistently or adequately met according to individual patient requirements (Kitson, 2016; Richards and Borglin, 2019). Researchers frequently point towards the finding that nurses, regardless of context, tend to ration fundamentals of care needs, such as mobilisation, toileting and bathing, skin and mouth care (personal care), and pain management, amongst others (Bail and Grealish, 2016; Ludlow et al., 2021). For older patients in need of complex care, an amalgamation of minor unfinished care activities can lead to serious functional decline and other severe complications. Nurses are, therefore, vital in assessing, evaluating, and preventing risk factors to avoid such complications. Consequently, nurses, here encompassing both registered nurses (RNs; bachelor’s degree) and non-RNs (healthcare workers or assistants), must possess the knowledge and skills to address the interplay between physical, psychosocial, and relational needs (Feo et al., 2018), while upholding their commitment to care (Kitson et al., 2013). This aligns with the broader scope of nursing practice (Royal College of Nursing, 2019), although it is particularly tied to the RN´s role (authorisation and responsibilities) and function (task and activities). These responsibilities involve critical thinking and the RN´s active engagement in clinical decision-making processes, most often grounded in the five steps of the nursing process: assessment, analysis, planning, implementation and evaluation of fundamentals of care needs (Wilkinson, 2011). Thus, the nursing process constitutes a vital part of nursing practice and its outcomes.

Addressing patients’ fundamentals of care needs aligns with Henderson’s (1997) seminal description of nursing practice, which involves assisting individuals in performing activities they would undertake independently, if possible, thereby fostering their independence (Henderson, 1997. p. 22). Effective nursing practice requires knowledge of the individual patient’s strength and abilities (Henderson, 1997). Adapting care to the patient’s functional level necessitates adjustments to care activities based on their resources, whether fully, partially, or in a compensatory way. Moreover, planning and implementing fundamentals of care activities often require collaboration with multidisciplinary teams, wherein nurses may operate dependently, interdependently, or independently (Wilkinson, 2011). The nurse’s performance level serves as a boundary for care activities within their remit and those delegated as interdependent tasks.

Delivering the fundamentals of care as the core of nursing is particularly important for older individuals (also referred to here as ‘residents’) in facility-based care. These individuals often have extensive care needs due to functional decline, multi-morbidity, and advanced age (Ng et al., 2020; Reilev et al., 2019). As a vulnerable group, the care provided to them should reflect high quality, particularly concerning fundamentals of care needs. Nursing practice is complex and can be understood as an ‘event’ within the larger healthcare system (Skivington et al., 2021). As a systems event, nursing practice is influenced by various contextual modulators. These modulators include individual factors, such as nurses’ dexterity, awareness, concentration, and decision-making abilities, as well as organisational factors, such as stress, fatigue, work environment, safety culture, communication, teamwork, and leadership (Lister et al., 2020). Additionally, organisational and cultural structure and leadership styles (Callen et al., 2009; Orvik, 2022) influence nurses’ attitudes and capabilities (Braithwaite et al., 2017; Cope and Murray, 2017). Understanding these contextual modulators is essential, as they affect the quality of care, defined as safe, effective, patient-centred, timely, efficient, and equitable (Institute of Medicine, 2001). Recent systematic reviews (cf. Nordaunet et al., 2024; cf. Savoie et al., 2023) indicate that few studies have yet examined nursing practice, its contextual modulators, and clinical decision-making simultaneously while targeting older residents’ fundamental care needs in a facility-based context such as a nursing home. At present, the majority of researchers investigating the framework have studied this issue in a hospital context. Consequently, there is limited understanding of to what extent nurses in a nursing home context engage in activities targeting older people’s fundamentals of care needs, the applicability of the framework, and what acts as contextual modulators. Furthermore, contextual modulators for practice require greater attention, especially within increasingly complex healthcare systems, where nursing practice should be studied as a part of a larger system. Thus, using the theoretical framework in exploratory research can support knowledge development, while evaluating the applicability of this framework to clinical nursing practice. Therefore, we aimed to explore nursing practice, its contextual modulators, and clinical decision-making, as aligned with the nursing processes, of nurses targeting older people’s fundamentals of care needs in nursing homes.

## 2 Methods

### 2.1 Study design

We employed an exploratory design (cf. Stebbins, 2008). Data (numerical and textual) were collected through structured direct observations (Bryman and Edward, 2019; Fix et al., 2022), supplemented by descriptive and reflective field notes (Patton, 2015). Numerical data were analysed using descriptive and comparative statistics and binomial general linear mixed models (GLMMs). Textual data were sorted and categorised based on similarities and differences.

### 2.2 Research context and setting

The Nordic countries are generally characterised by well-organised, accessible facility-based care for older people. In Norway, such care is mainly offered to older people with complex health care needs. Facility-based care, mainly provided in nursing homes, is predominantly municipally operated (91 %), with only 9 % provided by private healthcare organisations (Norwegian Directorate of Health, 2023). This reflects a division of care provision rooted in the principles of the Nordic welfare model, which emphasizes social rights and equality as vital components (Saunes et al., 2020). Regardless of the provider, all are statutorily responsible for meeting residents’ healthcare needs (Ministry of Health and Care Services, 2003). Access to quality healthcare provided by trained personnel is further regulated (Ministry of Health and Care Services, 2011), and nurses’ qualifications are specified by law or regulation (Saunes et al., 2020). No legal requirements for staff-resident ratios are defined (Saunes et al., 2020).

Three types of nurses, RNs, health care workers, and health care assistants, are present in the facility-based care context. Nowadays, the route into the nursing profession in Norway is via a bachelor’s degree in general nursing science. This involves 3 years of study at university college or at university. These studies result in both a professional foundation (licensure) and an academic degree. Specialist education (master´s degree in nursing science) is available for a variety of specialisations. The general RNs are equipped with the competencies, skills, and knowledge needed to take the lead of care for all patients, including those with complex and concurrent conditions (Ministry of Education and Research, 2019a). For health care workers (here non-RNs), the education is on the upper secondary school level, which, when completed, is followed by apprenticeship, trade certificate, and application for licensure (Ministry of Education and Research, 2019b). The health care assistants (here also non-RNs) are personnel lacking a relevant health-related education. In Norway, they are in a minority and are estimated to represent approximately 25 % of the nursing workforce (Flodgren and Meneses, 2017).

### 2.3 Sampling and recruitment strategies

**Fig. 1:**
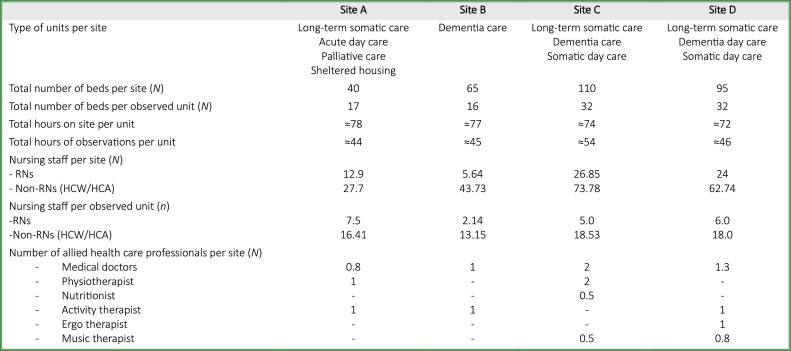
Contextual framing for data collection.

Abbreviations: RN=Registered Nurse HCW=Healthcare-Worker HCA=Healthcare Assistant.

We began by reviewing municipal and private nursing homes in southeastern Norway regarding location, size, and care focus. During the initial contact, two nursing homes declined participation due to resource and time constraints. Quality and municipal managers for the remaining six homes, along with service agencies in three municipalities, were contacted for approval. Two more nursing homes were removed to ensure optimal variation (Fig. 1). Ultimately, three public and one private nursing home unit (sites A–D) across three municipalities agreed to participate. Management at each site was informed via email and follow-up calls, with additional visits conducted. Recruitment posters with study details and contact information were displayed prominently, and a 2-day familiarisation visit preceded data collection. A purposeful recruitment technique (Emmel, 2013) was used to recruit the nurses and residents.

### 2.4 Data collection

Structured direct observations (Bryman and Edward, 2019; Fix et al., 2022) were conducted by the first author (OMN). Direct observations (e.g., using one’s senses by watching and listening) was used, as they are relevant when exploring the reality of practices without being a direct participant in the context. By structuring them (e.g., using an observational protocol), we could formulate explicit rules for what to observe and to record (Bryman and Edward, 2019). The first author was present for 12 consecutive days per site (A-D), resulting in a total of approximately 300 hours of field presence. To be able to observe as many fundamentals of care activities as possible, the first author, an RN, (OMN) followed the nurses’ rotas 7 days a week, while making sure to include both morning (07.30 – 15.30) and evening shifts (15.00 – 22.00) for the observations.

Two sampling techniques were used on site. The first, continuous sampling, involved observing the same nurse during a shift every time they engaged in fundamentals of care activities for residents. The other less-utilised method was instantaneous sampling, which involved observing and recording different nurses’ activities related to fundamentals of care activities at random intervals (Fix et al., 2022). Observations commenced when the nurses exhibited a cue (a signal, hint, or prompt in the protocol) to engage in a fundamentals of care activity (units of observations), and the observations ended when the nurse’s engagement in the activity was completed. The observations included both direct- and indirect fundamentals of care activities; i.e., resident part of the observed activity or resident not part of the observed activity. As our main study focus was on RNs’ scope of practice in relation to the fundamentals of care, the first author strived to recruit and observe 70 % RNs and 30 % non-RNs.

The structured direct observations were guided by an observational protocol developed by the first and last authors. It was initially tested during the first 2-day familiarisation visit that preceded data collection on site A. The protocol encompassed key areas based on established nursing concepts and frameworks. The primary area was the fundamentals of care framework (Feo et al., 2018; Kitson et al., 2013), covering physical (eight cues/items), psychosocial (eight cues/items), and relational (nine cues/items) needs, as well as the professional nurse-patient relationship (five cues/items). Additional areas included the nursing process, which is a model for nurses’ clinical decision-making (Wilkinson, 2011), performance, and functional levels (Henderson, 1997; Wilkinson, 2011), and contextual modulators of nursing practice (Lister et al., 2020), such as leadership styles (Cope and Murray, 2017), organisational structures (Orvik, 2022), and cultural influences (Callen et al., 2009). Data collection occurred between March and June 2024.

### 2.5 Data analysis

Numerical data were entered into IBM SPSS (IBM Corp., 2021). Descriptive and comparative statistics were utilised (Field, 2024). The Chi-square test (X^2^) was conducted to examine potential relationships between categorical variables, with significance set at *p* < 0.05. For binary outcomes over repeated measurements (nursing process - clinical decision-making model), binomial GLMMs in R were employed (R Core Team, 2023) to explore site variations and variations between the five steps in the nursing process. The model included sites A–D and the five steps as fixed effects, with random intercepts for individual RNs observed and for the different sites as random effects. The necessity of using random effects was confirmed via likelihood ratio tests for each response (degrees of freedom [*df*] = 5, *p* < 0.0001) and for both models. Comparative analysis across sites and steps was not possible due to insufficient observations related to the relational needs dimension of the framework. Descriptive field notes, comprising approximately 5400 words of descriptive content and 4700 words of reflective content, were read repeatedly, sorted, and categorised. These notes provided deeper insights beyond the numerical data.

### 2.6 Ethical considerations

We adhered to general research ethics regulations (All European Academies, 2023; World Medical Association, 2024) and the European Unions’ General Data Protection Regulation (Mondschein and Monda, 2018). The project was assessed by relevant authorities in Norway (Regional Ethics Committees ID 691021/2024 and the Norwegian Agency for Shared Services in Education and Research ID 524471/2024) and Sweden (Research Ethics committee, Karlstad University Sweden ID C2024/55 & Swedish Ethical Review Authority ID 2024–000586–01). All participants received written and oral information about the study before they signed a written consent. Participants was informed about their right to withdraw at any point without any further explanations. Proxy consent was obtained for residents not evaluated to have the capacity to give an informed consent. All data were securely transported and stored before being coded, anonymised, digitally curated, and uploaded to an encrypted file sharing service.

## 3 Results

A total of 411 observations (≈189 hrs) of nurses engaging in fundamentals of care activities were conducted by the first author. Each observation had a mean duration of 27.4 min (minimum 1 min; maximum 60 min). The nurses engaged in about 10.58 (mean) fundamentals of care activities per observation. Comparing time allocation per activity across sites revealed that observations at site A and B tended to be shorter than at site C and D. The two latter sites were additionally observed to engage in twice as many activities lasting 46–60 min, compared with sites A and B. No significant association was found between sites regarding the time taken by the nurses to complete the fundamentals of care activities they engaged in (X^2^ [9, *N* = 411] =12.79, *p* > 0.172) (Table 1).

**Table 1:** Descriptive characteristics of the observations.

Characteristics	Observations	Observations	Observations	Observations	Observations
	sites A–D	site A	site B	site C	site D
*Observations N* (%)	411	100 (24.4)	109 (26.5)	97 (23.6)	105 (25.5)
*Length of observations n* (%)					
- ≈ 1–15 minutes	135 (32.8)	36 (36.0)	42 (38.5)	24 (24.7)	33 (31.4)
- ≈ 16–30 minutes	135 (32.8)	34 (34.0)	33 (30.2)	30 (30.9)	38 (36.1)
- ≈ 31–45 minutes	74 (18.0)	18 (18.0)	22 (20.1)	20 (20.6)	14 (13.3)
- ≈ 46–60 minutes	67 (16.3)	12 (12.0)	12 (11.0)	23 (23.7)	20 (19.0)
*Shifts observed n* (%)					
- Monday – Friday	301 (73.2)	67 (67.0)	77 (70.6)	81 (83.5)	76 (72.3)
- Weekends/bank holidays	110 (26.7)	33 (33.0)	32 (29.3)	16 (16.4)	29 (27.6)
- Morning shift	349 (84.9)	78 (78.0)	90 (82.5)	91 (93.8)	90 (85.7)
- Afternoon shift	62 (15.1)	22 (22.0)	19 (17.4)	6 (6.10)	15 (14.2)
*FoC activities nursing staff engaged in n* (%)					
- FoC activities related to physical needs	874 (20.1)	232 (19.3)	208 (19.3)	200 (18.3)	234 (23.9)
- FoC activities related to psychosocial needs	1086 (25.0)	311 (25.9)	260 (24.2)	280 (25.6)	235 (24.0)
- FoC activities related to relational needs	1416 (32.5)	386 (32.1)	373 (34.6)	358 (32.7)	299 (30.6)
- FoC activities displaying a trusting nurse-patient relationship (commitment to care)	975 (22.4)	272 (22.7)	236 (21.9)	257 (23.4)	210 (21.5)
*Nursing staff part of observations n* (%)					
- Registered nurses	292 (71.0)	75 (75.0)	62 (56.8)	62 (63.9)	93 (88.5)
- Non-registered nurses	119 (29.0)	25 (25.0)	47 (43.1)	35 (36.1)	12 (11.5)
*Team composition in observations n* (%)					
- Working alone	283 (68.9)	77 (77.0)	79 (72.5)	55 (56.7)	72 (68.6)
- Working in pairs	111 (27.0)	23 (23.0)	24 (22.0)	36 (37.1)	28 (26.6)
- Team > 3 healthcare staff present	17 (4.10)	-	6 (5.50)	6 (6.20)	5 (4.80)
*Observations of direct and indirect FoC activities n* (%)					
- Registered nurses direct FoC activity	159 (38.6)	50 (50.0)	16 (14.7)	41 (42.3)	52 (49.5)
- Registered nurses Indirect FoC activity	132 (32.2)	23 (23.0)	45 (41.3)	23 (23.7)	41 (39.0)
- Non-registered nurses direct FoC activity	107 (26.0)	23 (23.0)	45 (41.3)	29 (29.9)	10 (9.50)
- Non-registered nurses indirect FoC activity	13 (3.20)	4 (4.00)	3 (2.70)	4 (4.10)	2 (2.00)
*Participants n* (%)					
- Older people – recipient of FoC activities	58 (42.0)	13 (40.6)	11 (33.3)	16 (47.1)	18 (46.2)
- Nursing staff	80 (58.0)	19 (59.4)	22 (66.7)	18 (52.9)	21 (53.8)
- Registered nurses	24 (30.0)	5 (26.3)	6 (27.3)	6 (33.3)	7 (33.3)
- Bachelor nursing students	5 (6.3)	-	1 (4.6)	4 (22.2)	-
- Non-registered Nurses – healthcare workers	34 (42.4)	7 (36.8)	12 (54.5)	5 (27.8)	10 (47.6)
- Non-registered nurses – healthcare assistants	17 (21.3)	7 (36.9)	3 (13.6)	3 (16.7)	4 (19.1)

**Abbreviations:**.

FoC = fundamentals of care.

*N*= total number.

*n* = *a* sample of the total number.

The observations indicated that nurses at sites A, B, and D primarily engaged in fundamentals of care activities alone, while nurses at site C more frequently worked in pairs. RNs at sites A, C, and D were observed participating in direct care activities (resident part of the observed activity) about half the time, whereas RNs at site B were mainly engaged in indirect care activities (resident not part of the observed activity). At site D, RNs allocated their time more evenly between direct and indirect care activities. A statistically significant difference was observed between sites in terms of the direct and indirect patient contact (X^2^ [3, *N* = 292] = 28.67, *p* < 0.001) (Table 1).

### 3.1 Nursing practice in relation to framework activities and functional levels

During the 411 observations, nurses engaged in 3376 activities across the three core dimensions of the framework: physical, psychosocial, and relational needs. Across all sites, most nursing activities fell within the relational needs dimension (41.9 %), followed by psychosocial needs (32.2 %), and physical needs (25.9 %) (Table 2).

**Table 2:** Descriptive overview of fundamentals of care activities observed.

Fundamentals of Care	Observations	Observations	Observations	Observations	Observations
(Kitson, 2013; Feo et al., 2017)	sites A–D	site A	site B	site C	site D
	(*N*=411)	(*n*=100)	(*n*=109)	(*n*=97)	(*n*=105)
*Observed FoC activity related to the dimension physical needs n* (%)					
-Numbers of observed activities:	874	232	208	200	234
- Rest and sleep	32 (3.66)	17 (7.3)	8 (3.8)	3 (1.5)	4 (1.7)
- Personal cleansing and dressing	102 (11.67)	22 (9.5)	23 (11.1)	25 (12.5)	32 (13.7)
- Medication management	238 (27.23)	58 (25.0)	58 (27.9)	51 (25.5)	71 (30.4)
- Toileting needs	97 (11.09)	28 (12.1)	16 (7.7)	23 (11.5)	30 (12.7)
- Eating and drinking	145 (16.59)	40 (17.2)	35 (16.7)	37 (18.5)	33 (14.1)
- Comfort including vital parameters, pain, breathing, and positioning	141 (16.13)	27 (11.6)	38 (18.6)	38 (19.0)	38 (16.2)
- Safety; e.g., risk assessment, risk prevention and hygiene	41 (4.69)	13 (5.6)	10 (4.7)	4 (2.00)	14 (6.0)
- Mobility	78 (8.92)	27 (11.7)	20 (9.5)	19 (9.5)	12 (5.2)
*Observed FoC activity related to the dimension psychosocial needs n* (%)					
-Number of observed activities:	1,086	311	260	280	235
- Verbal and written information adjusted to the patient	251 (23.1)	72 (23.2)	58 (22.3)	64 (22.9)	57 (24.3)
- Being involved and informed about the care	201 (18.5)	60 (19.3)	43 (16.5)	48 (17.1)	50 (21.3)
- Respected as a person	200 (18.4)	59 (19.0)	47 (18.1)	57 (20.3)	37 (15.7)
- Get education and information	53 (4.9)	12 (3.9)	12 (4.6)	10 (3.6)	19 (8.1)
- Values and beliefs are considered and respected	46 (4.3)	16 (5.1)	16 (6.1)	10 (3.6)	4 (1.70)
- Dignity protected, not being embarrassed or offended	175 (16.1)	54 (17.3)	43 (16.6)	43 (15.3)	35 (14.9)
- Emotional well-being, anxiety, worry or stress	114 (10.5)	30 (9.6)	30 (11.5)	31 (11.1)	23 (9.8)
- Right to privacy, not speaking above the patient	46 (4.2)	8 (2.6)	11 (4.3)	17 (6.1)	10 (4.2)
*Observed FoC activity related to the dimension relational needs n* (%)					
-Numbers of observed activities:	1,416	386	373	358	299
- Being emphatic, understanding the patient’s situation	250 (17.6)	71 (18.4)	58 (15.5)	65 (18.2)	56 (18.7)
- Helping patients to cope, supporting in developing and maintaining coping strategies	126 (8.90)	29 (7.5)	38 (10.2)	31 (8.7)	28 (9.4)
- Engaging with the patient, seeing them as individuals	205 (14.4)	59 (15.3)	54 (14.5)	52 (14.5)	40 (13.4)
- Supporting and involving family and carers	25 (1.8)	5 (1.30)	10 (2.7)	6 (1.68)	4 (1.33)
- Working with the patient to set goals	45 (3.2)	9 (2.30)	9 (2.41)	13 (3.6)	14 (4.7)
- Active listening, showing responsiveness	194 (13.7)	55 (14.2)	52 (13.9)	49 (13.7)	38 (12.7)
- Helping the patient to stay calm	169 (12.0)	41 (10.6)	48 (12.9)	42 (11.7)	38 (12.7)
- Being compassionate, show care for the patient	201 (14.2)	57 (14.8)	53 (14.2)	50 (14.0)	41 (13.7)
- Being physically and mentally present with the patient	201 (14.2)	60 (15.6)	51 (13.7)	50 (14.0)	40 (13.4)
*Observed FoC activity displaying commitment to care through the dimension a trusting nurse–patient relationship n* (%)					
-Numbers of observed activities:	975	272	236	257	210
- Developing relationship, inviting, introducing himself/herself, friendliness	262 (26.9)	72 (26.5)	61 (25.8)	69 (26.8)	60 (28.6)
- Focusing, eye contact, body language, interpreting signals	230 (23.6)	71 (26.1)	57 (24.2)	60 (23.3)	42 (20.0)
- Anticipating patients need before the emerge, reflecting prior experiences	225 (23.1)	62 (22.8)	55 (23.3)	60 (23.3)	48 (22.9)
- Knowledge about health history, fundamental needs, and the patients’ personal preferences	244 (25.0)	64 (23.5)	59 (25.0)	64 (24.9)	57 (27.1)
- Evaluating care in dialogue with patient	14 (1.4)	3 (1.10)	4 (1.70)	4 (1.70)	3 (1.40)

**Abbreviations:**.

FoC = fundamentals of care.

*n* = *a* sample of the total number.

Physical need activities (sites A–D) were focused on, presented in order of size, medication management, eating and drinking, and comfort, including monitoring vital signs, pain management, respiration and positioning (Table 2). The activity of medication management was observed 238 times out of the total number of different observed activities (*n* = 874) within this domain. The number of times the activity was observable within the different sites ranged from 51 at site C to 71 times at site D (range 20). Sites A and C showed higher engagement in eating and drinking activities than the other two sites. Safety-related activities, such as risk- assessment and prevention, accounted for 4.7–6 % of activities across all sites, with site C dedicating time to such activities only 2 % of the time (Table 2).

Psychosocial need activities largely comprised adjusting verbal or written information for the residents (presented in order of size), involving residents and informing them about care, and showing residents’ respect. Site D showed higher engagement in the activity adjusting information, involving residents and informing them about care. Activities related to respecting residents’ values, beliefs, and privacy were observed only 4.3 % of the time overall, occurring more often at site A and B but less frequently at site D (Table 2).

Relational needs activities typically involved being emphatic and understanding of residents’ situations (presented in order of size), engaging with residents as individuals and showing compassion, and being present (Table 2). Site A exhibited higher engagement in all these activities, whereas site D engaged less frequently. The least observed activities within this dimension were setting goals and supporting and involving relatives, observed only 3.2 % (*n* = 45) and 1.8 % (*n* = 25) of the time overall, with relatively small site variations (range 14 - 9 and 10 – 5 respectively) (Table 2).

For the final dimension of the framework, demonstrating commitment through a trusting nurse-patient relationship, 975 activities were recorded during the 411 observations. These included engaging in relationship building, knowing the resident, and interpreting residents’ signals. Activities such as interpreting signals varied from 26.1 % of the time at site A to 20.0 % at site D (Table 2). The analysis also showed that in 81.1 % of cases (362 observations), the main cue initiating the observation was the nurses addressing a physical need activity. Field notes elaborated on these numbers:

*Seven of nine residents supported out of bed and given morning care by the nurses [Physical needs: personal cleansing and dressing] before breakfast is served in the dining room [Physical needs: eating and drinking], medicines [Physical need: medication management] delivered at the table. Healthcare worker sits down with residents to support them to eat [Commitment to care: Being present, anticipation, and knowledge]. Table is set with fresh flowers and Easter decorations, soft music on radio in the background [Psychosocial needs: Dignity and values]*. (Descriptive Note #72, site A)

Comparing the number of activities nurses engaged in per observation across the framework’s dimensions revealed site-specific variations. In the physical needs dimension, the mean number of activities ranged from 1.9 to 2.06 at sites B and C, while nurses at sites D and A engaged in an average of 2.22–2.32 activities (mean variation: 0.42). For the psychosocial needs dimension, nurses engaged in an average of 2.64 activities per observation across all sites (A–D). However, greater variation was observed here compared to the physical needs dimension. The mean number of activities ranged from 2.88 at site B to 3.11 at Site A, while activities at sites D and C ranged from 2.23 to 2.38 (mean variation: 0.88). In the relational needs dimension, the average number of activities ranged from 3.69 to 3.86 at sites C and A, compared to an average of 2.84 to 3.42 at sites B and D (mean variation: 1.02). Regarding nurses’ commitment to care – defined as their efforts to establish a trusting nurse–patient relationship – the average number of activities observed ranged from 2.64 to 2.72 at sites C and A, while at sites B and D, the range was between 2.0 and 2.16 (mean variation: 0.72)

Observations related to residents’ functional levels showed that in 51.4 % of cases, care was *compensatory*; here the activity was wholly conducted by the nurses with no participation from the resident. *Partially compensatory* care occurred in 46.0 % of cases, where the activity was conducted in collaboration with the resident, even if the activity was led by nurse. In 2.6 % of the observation’s activities, were observed to be conducted as equal nurse – resident *collaborative care*. Compensatory care was most common in medication management, eating and drinking, and comfort-related activities, while collaborative care was more frequent in activities related to personal cleansing, dressing, and toileting needs. Descriptive notes highlighted these dynamics:

*RN takes order from resident who sits at table [Relational needs: active listening]. Nurse prepares breakfast [Physical needs: eating and drinking]. Medications served [Physical needs: medication management]. Resident to a lesser degree involved in preparing meal [Functional level: compensating]. Residents have mobility and strength to be more included in nutritional care. No visible attempt by RN to involve residents in meal preparation.* (Descriptive note #64, site A)

### 3.2 Nursing practice in relation to RNs’ clinical decision-making and performance level

**Fig. 2:**
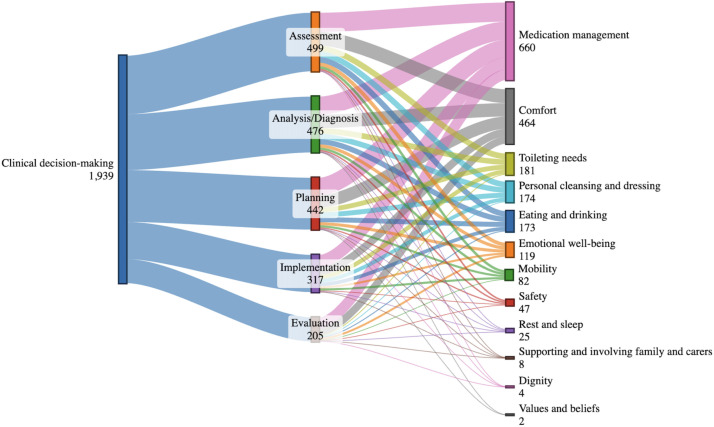
Registered nurses' clinical decision-making and the fundamentals of care.

RNs’ clinical decision-making, operationalised from the perspective of the steps in the nursing process, was observed in 292 instances, during which they engaged in 1939 fundamentals of care activities corresponding to different steps of the nursing process. Fig. 2 aims to visualise (Bogart, 2024) the complexity of the varying numbers of activities RNs engaged in across the five steps of the nursing process (i.e., decision-making model) (Fig. 2).

The variation across the steps of the nursing process – assessment, analysis, planning, implementation, and evaluation – in relation to the observed activities showed that the evaluation phase was less likely to occur compared to the first phase, assessment. This trend was evident for activities, such as personal cleansing and dressing (Odds Ratio [OR] 8.618, 3.672–20.230 Confidence Interval [CI], *p* < 0.001), medication management (OR 2.049, 1.517–2.768 CI, *p* < 0.001), toileting needs (OR 6.507, 3.049–13.878 CI, *p* < 0.001), eating and drinking (OR 6.323, 2.899–13.792 CI, *p* < 0.001), and comfort (OR 2.074, 1.495–2.878 CI, *p* < 0.001).

The model was also used to explore site-specific variations in the RNs’ engagement in the five steps of the nursing process, related to the different activities observed. We showed a statistically significant difference between sites regarding the physical needs dimension. RNs at site D displayed higher activity levels than those at site A (OR 0.51, 0.35–0.75 CI, *p* < 0.001) and site C (OR 0.61, 0.42–0.89 CI, *p* < 0.05). For activities related to psychosocial needs, site A showed lower engagement in the nursing process compared to site B (OR 0.02, 0.00–0.38 CI, *p* < 0.05), site C (OR 0.02, 0.00–0.39 CI, *p* < 0.05), and site D (OR 0.51, 0.00–0.36 CI, *p* < 0.01).

Observations of the RNs’ performance level, including their level of professional independence (autonomy) in activities, indicated independent, autonomous activities in 95.1 % (429 of 451 observed activities) of the observations focusing on physical needs. The most common activities observed were medication management (35.1 %) and comfort-related activities (21.8 %). For interdependent interventions (i.e., activities conducted in collaboration with other health care staff), 87.5 % were observed within the physical needs dimension.

### 3.3 Contextual modulators of nursing practice

Observations showed that all direct, individual modulators of nursing practice were present in 91.3 % (*n* = 1066 of 1168 possible) of observations of RNs’ activities (*n* = 292). The contextual modulator “ability to concentrate during activities” was observed in 223 of 292 observations, which means that RNs were interrupted during activities in 23.6 % (*n* = 69 of 292 possible) of observations (Table 3). These interruptions were described in the notes, as in the following example:

*RN interrupted [Direct factor: distracted] despite wearing signal yellow construction vest with print: ‘Do not disturb – I´m dispensing medicines’ [Physical needs and indirect modulator: safety culture].* (Descriptive note #81, site D)

**Table 3:** Contextual modulators of nursing practice.

Cues contextual modulators	Observations	Observations	Observations	Observations	Observations
(Lister et al., 2020; Cope and Murray, 2017)	sites A–D	site A	site B	site C	site D
	*N*=292	*n*=75	*n*=62	*n*=62	*n*=93
*Observed direct modulators (individual) RNs only n* (%)					
*-*Number of cues observed:	1,066	273	229	212	352
- Mental and physical dexterity/Yes	286 (26.8)	74 (27.1)	61 (26.6)	58 (27.4)	93(26.4)
- Ability to take note and assess the situation /Yes	281 (26.4)	73 (26.7)	61 (26.6)	58 (27.4)	89 (25.3)
- Ability to concentrate during activities/Yes	223 (20.9)	55 (20.2)	47 (20.6)	43 (20.2)	78 (22.2)
- Ability to make decision in a situation/Yes	276 (25.9)	71 (26.0)	60 (26.2)	53 (25.0)	92 (26.1)
*Observed potential modulators (organisational) RNs only n*(%)					
-Numbers of cues observed:	1,185	267	283	239	396
- No stress verbalised/Yes	280 (23.6)	72 (27.0)	62 (21.9)	56 (23.4)	90 (22.7)
- No fatigue verbalised/Yes	280 (23.6)	73 (27.3)	62 (21.9)	56 (23.4)	89 (22.5)
- Identifies and prevent risks/Yes	90 (7.7)	19 (7.1)	10 (3.5)	20 (8.4)	41 (10.4)
- Information transfer take place between personnel/Yes	168 (14.2)	27 (10.1)	48 (17.0)	39 (16.3)	54 (13.6)
- Ability to manage and lead nursing care/Yes	121 (10.2)	33 (12.4)	41 (14.5)	19 (7.9)	28 (7.1)
- Work environment positively supports RN in task/Yes	95 (8.0)	19 (7.1)	16 (5.7)	20 (8.4)	40 (10.1)
- Co-operation between personnel with different responsibilities)/Yes	151 (12.7)	24 (9.0)	44 (15.5)	29 (12.2)	54 (13.6)
*Transformational leadership styles (RNs only) n = times observed* (%)					
-Number of cues observed:	96	17	18	22	39
- Catalyst for change	5 (5.2)	1 (5.9)	1 (5.55)	-	3 (7.7)
- Democratic approach (share responsibilities)	64 (66.7)	13 (76.5)	11 (61.1)	18 (81.8)	22 (56.4)
- Goal-orientated (set clear expectations)	22 (22.9)	2 (11.7)	5 (27.8)	3 (13.7)	12 (30.8)
- Intellectual stimulators (influence to create and pursue new ideas)	1 (1.04)	-	1 (5.55)	-	-
- Inspirational	1 (1.04)	-	-	1 (4.54)	-
- Visionary (actively promote and articulate a vision)	3 (3.12)	1 (5.9)	-	-	2 (5.12)
*Transactional leadership styles (RNs only) n = times observed* (%)					
-Number of cues observed:	11	4	3	3	1
- Do not identify with shared values of the team	-	-	-	-	-
- Focus on managerial tasks	9 (81.8)	3 (75.0)	2 (66.6)	3 (100)	1 (100)
- Goal-oriented (tasks to be completed for reward)	-	-	-	-	-
- Lead change	-	-	-	-	-
- Make decisions quickly (effective in crisis)	2 (18.2)	1 (25.0)	1 (33.3)	-	-
- Motivational (provide rewards for the completion of tasks)	-	-	-	-	-

**Abbreviations:**.

RN = registered nurse.

*n* = *a* sample of the total number.

The analysis of 292 observations of RNs engaging in fundamentals of care activities also included cues for potential contextual modulators at the organisational level. Between five and seven modulators could be identified in 50.3 % of the observations. The three least observed cues for these modulators were identifying and preventing risks, work environment positively influencing nursing practice, and clearly observable RN leadership and management, represented in 25.8 % of observations (Table 3). Additionally, 24 observations (2.02 %) noted instances of RNs ‘omitting observable signs of stress or fatigue’, as exemplified in the following notes:

*Stressful shift for RN, few breaks, short lunch, seems exhausted as RN tries to stay on top [Potential cue for item: Show signs of stress and fatigue].* (Descriptive note #51, site D)

*Prior to conducting evening care, RN had a 5-minute break. Yawns, somewhat bloodshot eyes [Potential cue for item: Show signs of stress and fatigue].* (Descriptive note #27, site A)

The organisational structures at the sites were also evaluated. All sites met the criteria for hierarchical line management, with formal organisation, clear chains of command, administration management, and top-down leadership. Despite this, daily operations reflected a flatter structure, with vertical and horizontal cooperation and a flattened chain of command. The nurses were observed to exercise considerable autonomy in relation to activities relating to residents’ fundamentals of care needs.

In terms of organisational cultural styles during fundamentals of care activities, cues aligned with transformational leadership were present in 32.9 % of cases (96 of 292 observations). In 11 observations, cues for a transactional leadership style were detected. The observed organisational culture predominantly coincided with a constructive culture, wherein nurses were supportive, engaged in conflict resolution, constructive, and communicative and acted in the group’s best interest. The nurses were also observed to set challenging but realistic goals and pursue them with enthusiasm. However, rare instances of passive or defensive cultures were also observed, particularly in relation to oppositional behaviours, such as seeking status or influence by challenging others (Table 3).

## 4 Discussion

We aimed to explore nursing practice, its contextual modulators, and RNs’ clinical decision-making, as aligned by the nursing process, in addressing older people’s fundamentals of care needs in Norwegian nursing homes. To the best of our knowledge, it is the first study to observe nurses’ clinical practice in this context, using the fundamentals of care framework (Feo et al., 2018) as observational starting cues. Our exploration of contextual modulators supports the view of nursing practice targeting an integration of various fundamentals of care activities as an event in a complex system. Research on the framework is important, as unmet nursing care and failure to maintain are recognised as global challenges (Bail and Grealish, 2016; Chiappinotto et al., 2022), with fundamental care often delayed, partially completed, or omitted altogether (Bail and Grealish, 2016; Ball et al., 2014, 2018). Moreover, as Savoie and colleagues (2023) emphasised the lack of publications on fundamental care in the context of facility-based care, we therefor hope our findings can contribute to the scientific understanding of nursing practice targeting older people’s fundamentals of care in nursing homes. The following discussion focuses on the study’s key findings.

The framework, given its extensive theoretical and conceptual groundwork (Kitson et al., 2013), proved to be a reasonable reflection of nursing practice in the nursing home context. In our study, nurses were observed engaging in, as well as integrating, a variety of activities within the framework´s physical, psychosocial, and relational dimensions, but they were less often observed being involved in activities within the dimension of commitment to care, which emphasises a trusting nurse-patient relationship. The relative scarcity of activities in this dimension might stem from the challenges of observing certain cues, such as cognitive mindsets, or the inherent difficulty of internalising and translating these aspects into practice. The nurse-patient relationship consists of many underlying traits, from nonverbal communication, unconditional acceptance, and reciprocity (cf. Allande-Cussó et al., 2022). Thus, the complex relationship of traits makes the nurse-patient relationship arguably the most challenging aspect of the framework to observe. Moreover, the nurse-patient relationship can be understood as sensitive for contextual modulators as well. Consequently, lack of time to authentically engage, distractions, stress, and fatigue can in turn modulate the quality of authentic engagement (Pratt et al., 2021). Despite the nurse-patient relationship being at the very core of the framework (Feo et al., 2020), few tools exist that measure the nurse-patient relationship, reflecting the components that make up the core of the framework (Feo et al., 2022). Consequently, research on the framework in regard to the nurse-patient relationship in clinical nursing practice warrants further exploration in a variety of contexts and populations.

Nonetheless, we have provided insight into the nuanced nature of nursing practice in relation to the fundamentals of care. Despite the demanding work environment, we clearly demonstrated that nurses integrated the three dimensions in the framework into their practice. What started as a single fundamentals of care activity rapidly led to an amalgamation of care activities within the framework’s different dimensions. This adds further nuance to the complex practice of fundamentals of care (Muntlin et al., 2023) and the apparent integration of fundamentals of care activities in nurses’ practice, which is to lesser extent described in the scientific literature (Ottonello et al., 2023). To our knowledge, this pattern has not previously been described in more detail in relation to the framework. Literature reviews generally suggest the opposite: a strong nursing focus on physical needs and less so on psychosocial and relational needs (cf. Kalánková et al., 2021; Pentecost et al., 2020) or missed nursing care altogether (Grealish et al., 2023). Hence, future researchers on fundamentals of care should account for the integration of care, rather than focusing on single discrete activities described in the framework.

The importance of function-focused care (Resnick et al., 2013), particularly in the nursing home context, emerged clearly in our findings in relation to the functional level of nursing care activities (Henderson, 1997). We found that the nurses often compensated wholly or partially for residents’ fundamentals of care needs, with relatively modest involvement of residents in their care activities. Although many residents had lost functional autonomy, involving them in care activities still remains crucial to avoid reducing them to passive recipients of care. Shifting the focus from ‘doing for’ to ‘doing with’ aligns with a nursing practice that optimises or maintains older people’s functional abilities by evaluating their capabilities (McPherson et al., 2024; Resnick et al., 2013), rather than routinely defaulting to compensatory care. We suggest that models of care, such as function-focused care, should be implemented, especially as this model of care has been shown to be important in optimising quality of care (Resnick et al., 2024) and preventing adverse events due to failure of maintaining fundamental aspects of care (Bail and Grealish, 2016). Thus, research into nursing exploring the fundamentals of care framework while taking into account the residents’ functional ability level appears imperative to better understand the complex nuances of nurse’s scope of practice to develop a clearer picture of best practices.

RNs’ clinical decision-making, as aligned with the steps in the nursing process, appeared to focus primarily on assessment, rarely extending – going the distance – to evaluation of the fundamentals of care activities they were observed to engage in. This is notable, as evaluation is a central proficiency distinguishing RNs from non-RNs (Royal College of Nursing, 2019; Wilkinson, 2011). Our findings, unsurprisingly, align with others (Camargo-Figuera et al., 2021; Sheazeydi et al., 2024), highlighting the challenges RNs face in implementing all steps of the nursing process, leading them to prioritise assessment and implementation in their practice. RNs demonstrated a higher-level autonomy in fundamentals of care activities related to the physical dimensions, such as medication management and comfort (e.g., evaluating pain or the effects of antibiotics), which often involved all steps of the nursing process. In contrast, activities such as toileting, eating and drinking, and emotional well-being were less frequently addressed comprehensively. A plausible explanation for RNs’ lack of engagement in all the steps could be that non-RNs were based at the units, while RNs had, as expected, a more mobile role and function covering several units. Indeed, it is not uncommon for non-RNs to act as the ‘operational arm of RNs’ (Richards and Borglin, 2019, p.149), even though RNs’ responsibilities and activities, in this context, are ‘particularly crucial’ (Spilsbury et al., 2011, p. 747). This underscores the need for tools and resources to support quality fundamentals of care. Implementing the extended fundamentals of care framework, such as the fundamentals of care practice process (Feo et al., 2017), could help nurses apply a more stringent clinical reasoning process. Improving RNs’ evaluation of nursing care, particularly in relation to the fundamentals of care activities, remains essential. Not evaluating care activities can arguably lead to “failure to maintain” (Bail and Grealish, 2016), as older people´s fundamentals of care needs are not followed up. Equipping RNs with the necessary tools, such as digital health systems (Hants et al., 2023) and resources to support a comprehensive implementation of all the steps in the nursing process that underpins their decision-making, could enhance their roles in evaluation, supervision, and leadership in facility-based care.

Contextual modulators significantly influenced nursing practice targeting the fundamentals of care activities. Observations revealed individual and organisational modulators, such as inadequate risk management, a non-positive work environment, and unclear nursing leadership and care management. These issues, widely acknowledged in the literature (cf. Lister et al., 2020), affect care quality and safety, while also undermining care ethics and eroding patient care. Typically, evidence points towards lack of resources, heavy workload, and time pressure. However, leadership, (sub-)culture, organisational structures, and management are also crucial aspects modulating nursing care, as well as possible ethical dilemmas and workload. Nurses are often described as going “above and beyond” what they are required to do in order to maintain workflow (Waterfield and Barnason, 2022). Such task juggling could be tied to the observations as well, as nurses frequently went looking for things needed for a specific fundamentals of care activity or delivering multiple fundamentals of care activities simultaneously. This was especially the case for RNs, who were frequently interrupted or distracted in their work, despite distractions being associated with delayed care and lost concentration (Palese et al., 2018). Additionally, RN leadership was notably absent. Effective clinical leadership, as described by Cope and Murray (2017), is essential, as ‘all nurses are leaders’ (p. 61) and are expected to demonstrate leadership behaviours tied to professional comportment and care quality. Approaches, such as the Clinical Leadership Competency Framework (Nhongo et al., 2024)*,* that can support RNs to practice, maintain, and develop their leadership qualities, seem of importance, especially when RNs are expected to manage the complexities of care simultaneously with being confronted with a variety of contextual modulators and high care demands. Thus, implementing leadership frameworks, such as the Clinical Leadership Competency, could be one strategy in assisting RNs in developing a strong internalised professional identity; we know that the opposite can negatively influence nursing practice and the outcomes of nursing care (Goodolf and Godfrey, 2021; Godfrey, 2022). Lastly, the nursing homes, although hierarchical in theory, in practice seemed to adopt a flattened organisational structure. On one hand, the latter is described as stimulating communication, cooperation, and equality, while on the other hand, it can act as a double-edged sword, as it is also described as creating ambiguity, and role confusion (Essex et al., 2023). Regardless of organisational structure, nurses could benefit from clearly defined standards of proficiency (cf. Royal College of Nursing, 2019) and a balanced staff mixture (Peters et al., 2021). Such improvements can be beneficial while upholding the positive aspects associated with flattened organisational structures (Essex et al., 2023) to support RNs in their role and function as evaluators, managers, coordinators, leaders and planners of the fundamentals of care.

### 4.1 Methodological considerations

We took steps to ensure robust and reliable results. The exploratory design allowed us to gain insights into poorly understood phenomena, such as nursing practice in relation to the framework. First, the structured direct observations (Bryman and Edward, 2019; Fix et al., 2022), informed by established frameworks and theoretical concepts, enabled the collection of nuanced data on nursing practices and staff engagement in activities as they unfolded. Second, contextual understanding was a key focus, guided by the Medical Research Council’s framework for complex interventions (Skivington et al., 2021). Accordingly, our findings are relevant to contextual considerations and uncertainties in nursing practice. Third, attention to sampling (Emmel, 2013) and recruitment strategies, alongside pre-collection engagement with staff and residents, improved the quality of observations in a complex research context (Lam et al., 2018). Data were gathered through systematic, in-depth inquiry, generating both numerical and textual data, thereby enhancing the credibility of the results (Patton, 2015). The large number of observations collected over a prolonged period of time across multiple sites decreased the possibility of biased data. Personal involvements were minimised by limiting site visits and maintaining a non-participant role, observing unobtrusively, and interfering as little as possible (McNaughton et al., 2014), while balancing an outsider–insider position (Jootun et al., 2009). However, limitations of the study include data collection by a single researcher. The use of a structured observational protocol mitigated observer bias, but the Norwegian nursing homes involved in this study represent a small fraction of such facilities in Norway, requiring care in interpreting the findings. The limited geographic spread and reliance on one researcher’s observational cues should also be considered when evaluating the results.

## 5 Conclusion and implications

This is among the first studies to explore nursing practice targeting the fundamentals of care framework in nursing homes. We have highlighted the intricate nature of nursing practice and its relationship to decision-making, as aligned with the steps in nursing process, functional levels, and performance in nursing activities. Contextual modulators were found to significantly influence nursing practice, emphasising the need for improved working conditions for nurses. Intervention development research is warranted and should focus on optimising individual, organisational, and leadership modulators, as the improvement of these modulators can ultimately enhance patient safety and quality of care. Since this study provides initial evidence of how contextual modulators modify nursing practice targeting fundamentals of care in nursing homes, it is reasonable to assume similar effects in home-based care, a growing sector that also would gain from being researched in more detail. Finally, the study’s findings may inform the development of structured care models to support optimal nursing practice, particularly given the site-specific variations observed, which highlight the importance of approaches grounded in the fundamentals of care. The fundamentals of care framework can help nurses align their efforts and, alongside clearly articulated standards of proficiency, position them to fulfil their unique roles in facility-based care effectively.

## Funding

No external funding.

## CRediT authorship contribution statement

**O.M. Nordaunet:** Writing – review & editing, Writing – original draft, Visualization, Software, Project administration, Methodology, Investigation, Formal analysis, Data curation, Conceptualization. **E.R. Gjevjon:** Writing – review & editing, Supervision, Formal analysis, Conceptualization. **H. Aagaard:** Writing – review & editing, Supervision, Conceptualization. **C. Olsson:** Writing – review & editing, Supervision, Project administration, Conceptualization. **G. Borglin:** Writing – review & editing, Writing – original draft, Supervision, Project administration, Methodology, Investigation, Formal analysis, Conceptualization.

## Declaration of competing interest

The authors declare that they have no known competing financial interests or personal relationships that could have influenced the work reported in this paper.

## Data Availability

The raw data that supports the findings of this study are not available due to confidentiality and ethical restrictions including data would not be shared. The developed and tested observational protocol are available on request from the corresponding author.
